# Machine learning predicts live-birth occurrence before in-vitro fertilization treatment

**DOI:** 10.1038/s41598-020-76928-z

**Published:** 2020-12-01

**Authors:** Ashish Goyal, Maheshwar Kuchana, Kameswari Prasada Rao Ayyagari

**Affiliations:** BML Munjal University, Gurugram, 122413 India

**Keywords:** Computational biology and bioinformatics, Origin of life

## Abstract

In-vitro fertilization (IVF) is a popular method of resolving complications such as endometriosis, poor egg quality, a genetic disease of mother or father, problems with ovulation, antibody problems that harm sperm or eggs, the inability of sperm to penetrate or survive in the cervical mucus and low sperm counts, resulting human infertility. Nevertheless, IVF does not guarantee success in the fertilization. Choosing IVF is burdensome for the reason of high cost and uncertainty in the result. As the complications and fertilization factors are numerous in the IVF process, it is a cumbersome task for fertility doctors to give an accurate prediction of a successful birth. Artificial Intelligence (AI) has been employed in this study for predicting the live-birth occurrence. This work mainly focuses on making predictions of live-birth occurrence when an embryo forms from a couple and not a donor. Here, we compare various AI algorithms, including both classical Machine Learning, deep learning architecture, and an ensemble of algorithms on the publicly available dataset provided by Human Fertilisation and Embryology Authority (HFEA). Insights on data and metrics such as confusion matrices, F1-score, precision, recall, receiver operating characteristic (ROC) curves are demonstrated in the subsequent sections. The training process has two settings *Without feature selection* and *With feature selection* to train classifier models. Machine Learning, Deep learning, ensemble models classification paradigms have been trained in both settings. The Random Forest model achieves the highest F1-score of 76.49% in *without feature selection* setting. For the same model, the precision, recall, and area under the ROC Curve (ROC AUC) scores are 77%, 76%, and 84.60%, respectively. The success of the pregnancy depends on both male and female traits and living conditions. This study predicts a successful pregnancy through the clinically relevant parameters in In-vitro fertilization. Thus artificial intelligence plays a promising role in decision making process to support the diagnosis, prognosis, treatment etc.

## Introduction

More than 80 million couples are affected by infertility. An 'unsuccessful conception' after almost 12 months of having unprotected intercourse can be caused due to infertility^1^. To reduce the number of unsuccessful conception, ovum from the female ovary and sperm from the male are fused outside the body, i.e., in the laboratory resulting in an embryo, which is then placed in the female's ovary for development is In-Vitro Fertilization (IVF). In some cases, artificial insemination results in conception by injecting sperm into the uterus directly. It has been reported that more than 5 million babies have been born from IVF around the world. IVF is used to overcome the male and female infertility caused due to various problems related to both the sexs' reproductive characteristics. IVF works by combining various medical and surgical procedures to help in fertilization. The whole process has more than one round and can take several months to get a pregnancy. Easy accessibility of IVF treatments rose the usage of IVF but not due to infertility couples^2^. Opting for IVF treatment is also considered a very challenging task due to its high cost, no guarantee of the success, and the stress of the treatment^3,4^. Patients generally discontinue IVF treatment due to the physical and psychological burden of the treatment^5,6^.

Several medical practitioners have been predicting the possibility of pregnancy by a trial and error method through their expertise. Therefore, conventional prediction methods are dependent on the level of experience of an individual medical practitioner, which does not employ any systematic statistical approach. Hence, they are more subjective. Medical practitioners and patients are eagerly looking for a measurement to guide them for decision making about IVF treatment. Recent advancements in technologies such as Artificial Intelligence (AI), Machine Learning (ML), Deep learning (DL) promises in solving some of the endemic problems with statistical data-driven approaches. Highly accurate analysis driven by AI can lead to radically solve most of the challenges with vast amounts of data by interpreting them in a meaningful way. The statistical approach has attracted researchers' attention to develop prediction models for fertility by which a medical practitioner can get an accurate prediction if a successful birth happens in the IVF setup.

Machine Learning is a field of study that teaches computers/systems to think in a similar way and outputs predictions by learning/training upon past experiences^7^. It explores data in a meaningful, pattern-oriented manner that gives the systems' robustness to mimic a human decision-making capability. Deep learning is a subset of ML that works on the principles of human neural networks^8^. Analyzing huge data records with lots of parameters can make humans miss some crucial patterns in the data. ML and DL get you covered in this aspect and can identify these patterns easily, which then helps a human in decision-making.

With continued improvements in ML, several healthcare domains have already adopted it to improve decision-making, including enabling personalized care, surgery simulations, drug discovery, and accelerating disease diagnosis^9–12^. In reproductive science, ML was applied for predicting implantation after blastocyst transfer in IVF^13^. ML has also been applied to build a prediction model for embryo selection to evaluate the live-birth live-birth predictors and predict twins^14–16^. Contemporary use of DL techniques to predict fatal heart pregnancy and human blastocyst selection have also been witnessed^17,18^. Hence, ML can be applied to the clinical datasets to develop risk assessment, diagnostic, prognostic models, and improve patient healthcare.

Machine Learning Some studies in the past use ML techniques to predict the live-birth chances of women undergoing IVF treatment. One of the earlier and most accepted prediction models is the McLernon Model^19,20^, which utilizes only discrete logistic regression to predict the chances of live-birth for a couple having up to six complete IVF cycles. Two prediction models, a pre-treatment model (predicts before the IVF treatment starts) and a post-treatment model (predicts the chances of live-birth after the first attempt at embryo transfer), are developed. Data was collected from The Human Fertilisation and Embryology Authority (HFEA) of 253,417 women who started IVF treatment in the United Kingdom from 1999 to 2008 using their own eggs and partner sperms. C-index was used to assess the performance of both models. The C-index for pre-treatment model was 0.69 (0.68–0.69) and C-index for post-treatment model was 0.76 (0.75–0.77).

Rafiul Hassan et al.^21^ proposes a hill-climbing feature selection algorithm with five different ML models to analyze and predict IVF pregnancy in greater accuracy. Data for this study were collected from an infertility clinic in Istanbul, Turkey, for about three years from March 2005 to January 2008 and consists of infertility treatment of 1048 patients. This study used 27 attributes like age, diagnosis, Antral Follicle Counts (AFC), sperm quality, etc. It is found that age is the most influential IVF attribute that affects pregnancy outcome. Performance of all classifiers improved when hill climbing feature selection techniques (electing only important features by the classifiers) was employed. Overall, Support Vector Machine (SVM) attains the highest accuracy of 98.38%, F1-score of 98.4%, and AUC score of 99.5% considering 19 IVF attributes.

A survey done by Guvenir et al.^22^ on the ML models, namely SVM, Decision trees, Naïve Bayes, K nearest neighbor (KNN), etc. showed that different models require a various number of features to perform well. Patient attributes such as age, Body Mass Index (BMI), sperm count, etc. were used to train these models. SVM in this survey considers up to 64 features to resulting in an accuracy of 84%, while others considered as low as 5–6 features like artificial neural networks (ANN) by Kaufmann et al.^23^ that resulted in 59% accuracy^24^. focuses on developing a model that helps the couple decide whether to take IVF treatment or not. In this survey, two problems are addressed: one to check the probability of having pregnancy in the IVF treatment and another in helping doctors choose the most viable embryos.

Jiahui Qiu et al.^25^ predicts live birth before the IVF using four models: logistic regression, Random forest, Extreme gradient boosting (XGBoost), and SVM. Data is collected from 7188 women who underwent their first IVF treatment from the Medical Center of Shengjing Hospital of China Medical University during 2014–2018. Attributes like age, AMH, BMI, duration of infertility, previous live birth, previous miscarriage, etc. and type of infertility (tubal, male factor, anovulatory, unexplained, and others) are considered. Calibration and receiver operating characteristic (ROC) curves are employed as performance metrics. XGBoost achieved the highest area under ROC curve (ROC AUC) score of 0.73 on the validation dataset and exhibited the best calibration model among all models.

Predicting the live-birth occurrence belongs to the binary classification problem determining whether a female gives birth or not is predicted based on the given IVF parameters. The present work aims to compare various models on predicting live-birth occurrence after the complete IVF cycle. The work mainly focuses on making predictions of live-birth occurrence when an embryo forms from a couple and not a donor. A complete IVF cycle refers to the fresh cycle and the following freeze–thaw cycles from one round of ovarian stimulation. There are several reproductive characteristics related to both males and females that cause infertility. It has been found that factors related to female-like age (decrease in quantity and quality of the eggs), menstrual disorder, uterine factor, cervical factor, previous pregnancies, duration of infertility, female primary (if the patient is unable to get pregnant after at least one year), female secondary (if the patient able to get pregnant at least once but now unable to) and unexplained factors have a significant impact on causing infertility^26–30^. Factors related to males, such as semen concentration, semen motility, semen morphology, sperm volume, and semen count, are essential for testing infertility in males^31,32^. All the above-said important reproductive characteristics of males and females have been considered in this study. A public dataset^33^ that contains all the above-said parameters, provided by Human Fertilisation and Embryology Authority, is the longest-running fertility treatment database in the world. Data of 495,630 records with 94 clinical features are considered in this study acquired from 2010 to 2016 from IVF centers across the UK. After performing data cleaning 141,160 records with 25 essential clinical features are considered for training and testing in which both positive and negative classes contain 70,580 records each.

ML, DL, Ensemble learning are employed in this study. Models such as Logistic Regression^34^, K nearest neighbor^35^, Multi-Layer Perceptron^36^, Decision Tree^37^, 1-D Deep learning model^38^ are used for training purpose. Ensemble learning^39^ is used to make a collective decision on predictions from the above-said models. Random forest^40^, AdaBoost^41^, voting classifiers^42^ are employed in this strategy. Predominantly, two settings are followed in this study: one training model without feature selection and the other using two feature selection techniques. Feature selection techniques such as Linear support vector classifier (Linear SVC) based selection from model^43^, Tree-based feature selection^44^ are considered. The performance of models in both these settings was measured based on metrics such as F1-score, precision, recall, and ROC-AUC curve.

The remainder of this article is organized as follows: in the Methodology section describes the dataset, pre-processing techniques used to make train-ready data and models trained. A comprehensive comparison between the performance metrics of various models is demonstrated in Results & Discussion. The conclusion section explains about the insights that can be drawn and future work from this study.

## Methodology

### Dataset description

The dataset used in this study is anonymized register data collected from the year 2010–2016 obtained from the Human Fertilisation & Embryology Authority^33^. It holds the longest-running database register of fertility treatments globally to improve patient care while ensuring reliable protection of patient, donor, and offspring confidentiality. This dataset contains 495,630 patient records with 94 features on treatment cycles collected from various patient studies between 2010 and 2016. Focusing on the aim of this study, the dataset after filtration contains 141,160 patient records. It includes three types of data that are numerical, categorical, and text. *There was no medical intervention in the couples' behavioral and biomedical routine for this work. Furthermore, this work involves only analyzing the couples' data; hence no permission was taken from the Institutional Review Board (IRB). All relevant guidelines were followed for the study.*

Many factors influence the live-birth occurrence (target variable) after IVF treatment. In this study, the original dataset contains 94 features, but not all features significantly affect the outcome. So, only 30 features are considered. Hence, feature engineering has been performed based on the subject knowledge, recommended by Dr. Bharti Bansal and NICE clinical guideline (The National Institute of Health and Care Excellence, UK). In this study, features such as fresh cycles and the following freeze–thaw cycles from the same stimulation for a woman undergoing IVF (including ICSI) are considered. Donor oocytes/sperm cycles and PGD/PCS cycles are excluded. Features selected in this study are age, the total number of previous cycles, the total number of previous IVF pregnancies, number of eggs mixed with partner sperm, number of embryos transferred in this cycle, type and cause of infertility that includes male factor, female factors, ovulatory, endometriosis, tubal, cervical, etc. Table 1 summarizes a detailed description of the dataset features considered in the approach.

**Table 1:** IVF attributes of our dataset.

Field	Type	Description
Patient age at treatment	Categorical	Patient age at treatment, banded as follows: 18–34, 35–37, 38–39, 40–42, 43–44, 45–50
Total number of previous cycles	Numerical	How many treatment cycles of IVF the patient has previously had
Total number of IVF pregnancies	Numerical	How many patients have been pregnant through IVF
Total number of live births- conceived through IVF	Numerical	How many live births the patients have had through IVF
Type of infertility—female primary	Categorical	1 if the patient unable to get pregnant after at least 1 year, 0 otherwise
Type of Infertility—female secondary	Categorical	1 if the patient able to get pregnant at least once but now unable to, 0 otherwise
Type of infertility—male primary	Categorical	1 if the leading cause of the infertility is patient, 0 otherwise
Type of infertility—male secondary	Categorical	1 if the secondary cause of infertility is due to the patient, 0 otherwise
Type of infertility—couple primary	Categorical	1 if the leading cause of the infertility is patient/partner, 0 otherwise
Type of infertility—couple secondary	Categorical	1 if the secondary cause of infertility is due to the patient/partner, 0 otherwise
Cause of infertility—tubal disease	Categorical	1 if there is damage in the fallopian tubes that prevents sperm from reaching the ovary, 0 otherwise
Cause of infertility—ovulatory disorder	Categorical	1 if the primary cause of this infertility is due to ovulation disorder, 0 otherwise
Cause of infertility—male factor	Categorical	1 if the primary cause of this infertility is due to male patients, 0 otherwise
Cause of infertility—patient unexplained	Categorical	1 if the primary cause of infertility in the patient is unknown, 0 otherwise
Cause of infertility—endometriosis	Categorical	1 if the primary cause of this infertility is due to endometriosis, 0 otherwise
Cause of infertility—cervical factors	Categorical	1 if the primary cause of this infertility is due to the Cervical factor, 0 otherwise
Cause of infertility—female factors	Categorical	1 if the primary cause of this infertility is due to female factors, 0 otherwise
Cause of infertility—partner sperm concentration	Categorical	1 if the primary cause of this infertility is due to low sperm count, 0 otherwise
Cause of infertility—partner sperm morphology	Categorical	1 if the primary cause of this infertility is an abnormality in sperm morphology, 0 otherwise
Cause of infertility—partner sperm motility	Categorical	1 if the primary cause of this infertility is poor sperm motility, 0 otherwise
Cause of infertility—partner sperm immunological factors	Categorical	1 if the primary cause of this infertility is due to sperm immunological factors, 0 otherwise
Stimulation used	Categorical	1 if the stimulation medication is used, 0 otherwise
Egg source	Text	Indicates whether the eggs used in this cycle came from Patient (P) or a Donor (D)
Sperm source	Text	Indicates whether the eggs used in this cycle came from Patient (P) or a Donor (D)
Fresh cycle	Categorical	1 if this cycle using fresh embryos, 0 otherwise
Frozen cycle	Categorical	1 if the cycle used from frozen embryos, 0 otherwise
Eggs thawed	Numerical	If this cycle frozen eggs, the number of eggs thawed
Fresh eggs collected	Numerical	The number of eggs collected in this cycle
Eggs mixed with partner sperm	Numerical	The number of eggs mixed with sperm from the partner
Embryos transferred	Numerical	The number of embryos transferred into the patient in this cycle

### Pre-processing of dataset

The raw dataset contains 94 attributes, out of which few do not significantly affect predicting live-birth occurrence. The filtration of the dataset depends on the stimulation used, sperm source, egg source features. If the source of sperm and egg is from the same couple, i.e., Partner and Patient, then those patient records are considered, the rest are eliminated. In IVF, injectable medication containing both follicle-stimulating hormone (FSH) and luteinizing hormone (LH) is injected into females to stimulate more than one egg developing at a time^45^. It is described as "Stimulation Used" in the dataset; this study considers only patient records where stimulation is done.

In the field "Patient Age at Treatment," few patient records contain value 999 that are eliminated. Text and age ranges are converted into categorical data. For instance, in the field "Patient Age at Treatment."

1. 18–34 is converted to 0
2. 35–37 is converted to 1
3. 38–39 is converted to 2
4. 40–42 is converted to 3
5. 43–44 is converted to 4
6. 45–50 is converted to 5

The field "Live-birth Occurrence" is the target variable, which is numerical and contains values ranging from 0 to 5 where 0 represents no birth (negative class) and greater than 1 represents birth occurrence (positive class). To make the classification binary, all the patient records whose Live-birth Occurrence are more significant than 1 are set to 1 and remaining to 0.

After the above filtration, the negative samples are 5 × more than positive samples that make data imbalance. Few patient records from negative samples are removed to encounter the problem of imbalance in the dataset. Now the dataset contains 141,160 patient records and 25 features, which distributes 70,580 samples in each class. It is found that few fields such as sperm source, egg source, cause of infertility partner sperm immunological factors, stimulation used, cause of female infertility factors is homogenous (contains same value) in both positive and negative cases, which adds up no significance in classification hence these fields are removed. The samples or patient records are then split by 34% in the validation and 66% in training sets.

- The training set contains 93,165 samples
- The validation set contains 47,995 samples

**Figure 1:**
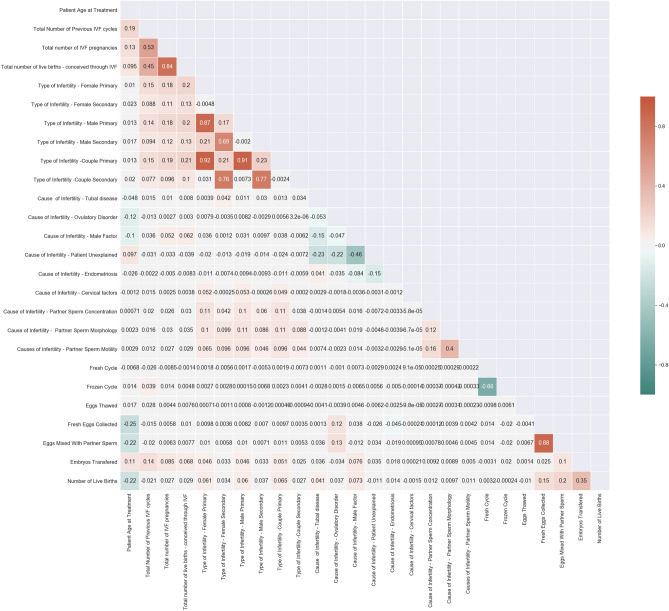
Correlation matrix of 25 features.

Data is normalized as it contains values which are distributed in an extensive range of integers. Features with very similar trends are also likely to carry very similar information. In this case, only one of them will suffice to feed the Machine Learning model. A correlation matrix constructed is shown in Fig. 1 contains 25 features that reveal the importance of each of the parameters on the model developed. Here we calculate the correlation coefficient between numerical and nominal columns as the Coefficient and the Pearson's chi-square^46^ value. Pairs of columns with a correlation coefficient higher than a threshold are reduced to only one.

### Model training

#### Overview

In this study, Machine Learning , Deep learning, and ensemble models are trained for the purpose. Machine Learning ML models such as Logistic Regression (LR), K nearest neighbor (KNN), Multi-Layer Perceptron (MLP), Decision Tree are used for training. A Deep learning model, especially a 1-D Neural Network with a sigmoid activation neuron at the output layer, is proposed. Ensemble learning is also employed to get a concrete decision from a list of Machine Learning models. Random Forest, AdaBoost, Voting classifier hard/soft are considered in ensemble learning techniques.

#### Two settings

**Figure 2:**
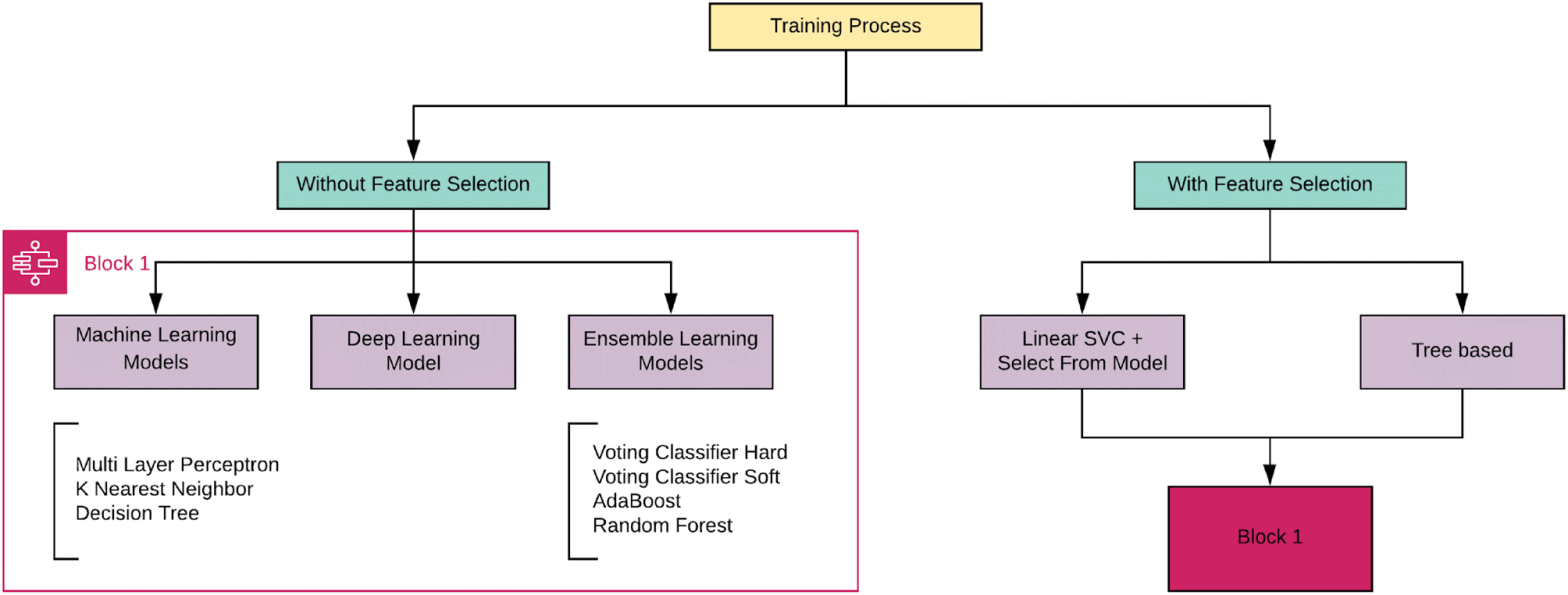
Flowchart of the training process.

Training models in this study takes place in two settings, one With Feature Selection and the other Without Feature Selection. Feature selection techniques are applied to get the essential features from the dataset. In the Without Feature Selection setting, all the features (25 in total) are used in the training process. In the With Feature Selection setting, only certain features (important ones) are used in the training process based on the technique. This approach gives a comprehensive analysis of results where models have been trained on data with and without feature selection. A flowchart of the training protocols employed in this study is detailed in Fig. 2. The models trained under *With feature selection & Without feature selection* setting remain the same as explained in Fig. 2.

After taking suggestions from domain expert Dr. Bharti Bansal we selected the essential features, but statistical feature importance is employed later. Hence exploring different feature selection algorithms may help a lot in improving overall performance factors. Feature selection techniques selected are

1. Linear support vector classifier (Linear SVC) + Select From Model
2. Tree-based feature selection

#### *Linear SVC* + *Select From Model*

Linear models penalized with the L1 norm have sparse solutions: many of their estimated coefficients are zero. If the goal is to reduce the dimensionality of data and use another classifier, they can be used along with *feature_selection.SelectFromModel* in the scikit-learn to select the non-zero coefficients. Sparse estimators useful for this purpose are the Lasso for regression, Logistic Regression, and Linear SVC^43^. The sparse estimators used in this method are Logistic Regression, Decision Tree, Random Forest, K Nearest Neighbours classifier. The feature space reduces from 25 to 20 using this technique.

#### *Linear SVC* + *Tree-based feature selection*

Tree-based estimators such as Random Forests, once trained, the importance of each feature is computed with which we can filter and reduce the feature space. Every feature in random forests, while training is given with a Gini impurity or information gain/entropy using this measure, we calculate the feature importance^44^. After reducing the feature space, we can then train different estimators or classifiers on this new set. This study's sparse estimators are Logistic Regression, Decision Tree, Linear Discriminant Analysis, Random Forest, K Nearest Neighbours. The feature space reduces from 25 to 5 using this technique.

#### Deep learning: custom deep neural network

Along with the ML models, a Deep learning classifier (DL) architecture was trained on the same data. The neural network takes numerical values (array of size 25) as the input; hence it is 1-dimensional in the architectural perspective (1-D Model). The output layer contains one neuron with a sigmoid activation function to give a binary output (Birth occurrence or Not). The architecture contains a total of 9 dense layers, each neuron (in all dense layers) output values are passed through a Rectified Linear Unit (ReLU)^47^ activation function. In the first half of the DL classifier, neurons in each layer get increased precisely two times the previous layer; this is maintained uniform because of performance on this dataset. The second half follows a decreasing rate of two neurons per layer, making the last layer one. Adam optimizer^48^ is used for optimizing loss values while training the deep neural network. Due to its broader adoption in Deep learning applications and combining the AdaGrad and RMSProp algorithms' best properties to provide an optimization algorithm that can handle sparse gradients on noisy problems, Adam optimizer is chosen. Not just on the theory intuitions of Adam optimizer's performance, but also the performance on this dataset is checked across different optimizer algorithms such as Stochastic gradient descent, RMSProp, AdaGrad, and it is noticed that Adam optimizer performance is better than others.

**Figure 3:**
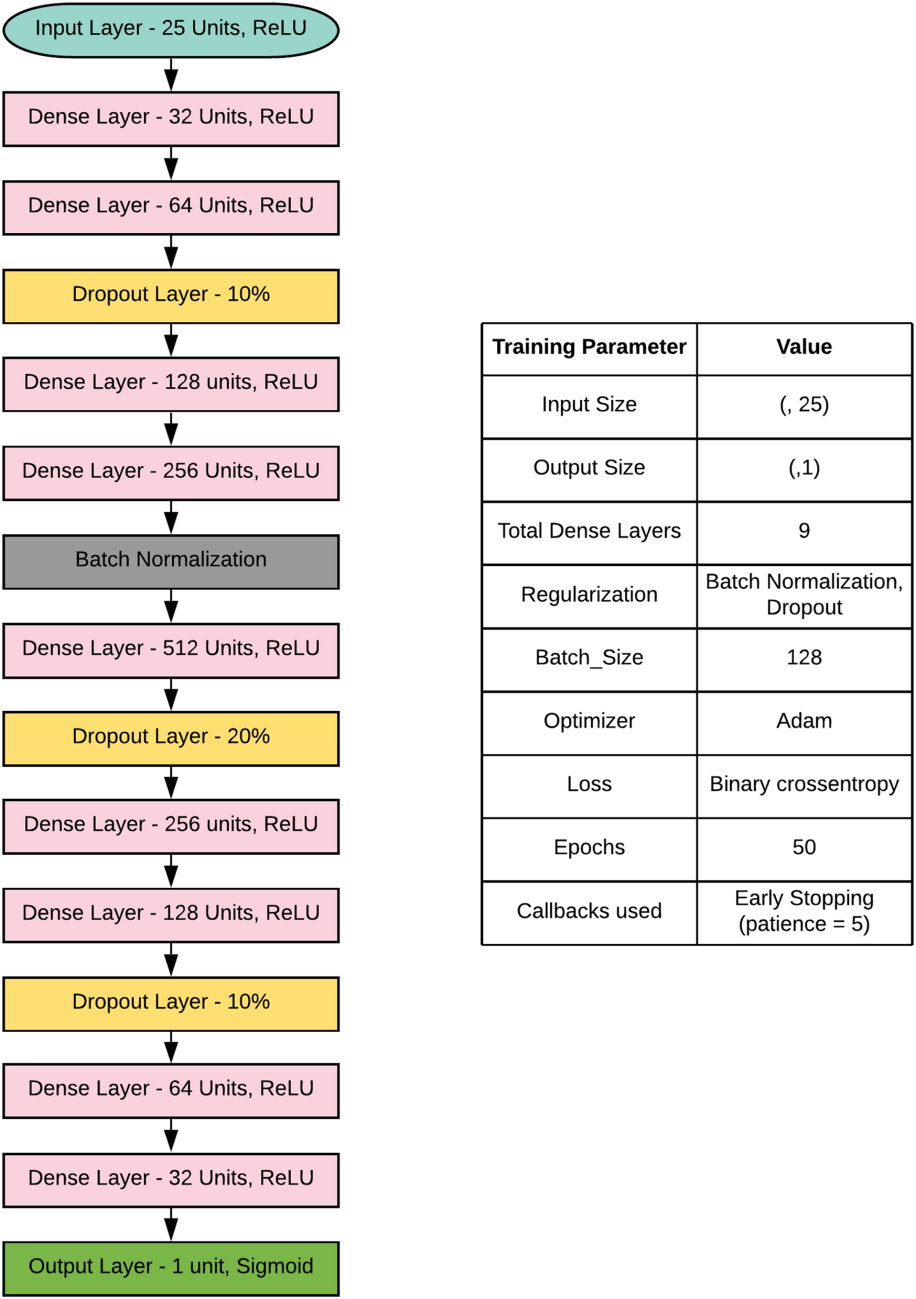
Deep learning architecture, along with the training parameters explained.

The total number of epochs to train the DL classifier is 50. The model can overfit this dataset to prevent overfitting regularization techniques such as Dropouts and Batch Normalization^49^, Early stopping has been employed while training. As the number of neurons increases, the probability of noise generation will be higher among dense middle layers of the DL classifier, so 20% of dropouts are introduced after the middle-dense layer (512 units). Binary cross-entropy loss fits best for binary classification set up when trained on Deep learning techniques. Computing the gradient over the entire dataset is expensive, and hence batch size of 128 samples has been trained per epoch to get a reasonable approximation of the gradient. A glimpse of custom deep learning architecture is depicted in Fig. 3.

#### Ensemble learning

Ensemble methods are the most famous learning algorithms in the ML domain because of its excellent performance. The whole crux of these methods is that it combines many ML algorithms to make an accurate decision. In this study, the following algorithms are trained.

1. Random Forest
2. AdaBoost
3. Voting classifier—soft/hard

#### Voting classifier

It is a wrapper of many classification models where the final decision is made by voting from individual models' predictions. For instance, if five binary classification models are trained and queried with an unseen sample, then the individual model's predictions are input for the voting system, declaring final prediction. Supplemental Fig. 1. illustrates a voting classifier.

The voting system works on two different strategies: Hard Voting, Soft Voting. Hard voting is also called the Majority voting in which the class that gets the highest number of votes from a set of individual models is selected^42^. If **Nc** is the number of votes for a class and **y1**,** y2**,** y3**, ….,** yn**, are predictions of **n** different classifiers, then the hard-voting formula is given by Eq. (1).

1 $$y_{final} = Max\left( {N_{c} \left( {y_{1} } \right),N_{c} \left( {y_{2} } \right),N_{c} \left( {y_{3} } \right), \ldots ,N_{c} \left( {y_{n} } \right)} \right)$$

Soft voting takes input as probability scores vector from individual class and sums it with all other classifiers later averages it^42^. The final output class will be the one that gets the maximum probability score. If **p**_**1**_,** p**_**2**_, …,** p**_**n**_ are the probability scores of **n** different classifiers, then the formula for soft voting is given by Eq. (2).

2 $$y_{final} = Max\left( {\frac{1}{n}\left( {\sum {\left( {p_{1} ,p_{2} , \ldots ,p_{n} } \right)} } \right)} \right)$$

The classifiers used for this voting classifier are Logistic Regression, Decision Tree, Linear Discriminant Analysis, Random Forest, and K Nearest Neighbours. In the next section, the model has been validated with experimental datasets.

## Results and discussion

In this study, the TensorFlow library with Keras backend to train deep learning classifier and scikit-learn for the ML classifiers are utilized. The metrics compared in this study are F1-score, precision, recall, ROC AUC scores, and curves between various models. In this section, we demonstrate the results of trained models *With* and *Without feature selection*. In comparison tables, broader categories are displayed, such as ML-based, DL-based, Ensemble-based. Table 2 details the comparison between trained models *Without feature selection*.

**Table 2:** Comparison between classification metrics for without feature selection models.

	Model	Precision (%)	Recall (%)	F1-score (%)	ROC AUC score (%)
Machine learning models	Multi-layer Perceptron	74	72	72.98	77.90
	K Nearest Neighbours	71	71	71.00	77.60
	Decision Tree	76	76	76.00	83.30
Deep learning model	DL Classifier	73	72	72.49	78.00
Ensemble Learning models	Voting—hard classifier	75	73	73.98	73.10
	Voting—soft classifier	77	75	75.98	83.20
	Random forest	77	76	76.49	84.60
	AdaBoost	74	72	72.98	77.40

### Results for without feature selection setting

**Figure 4:**
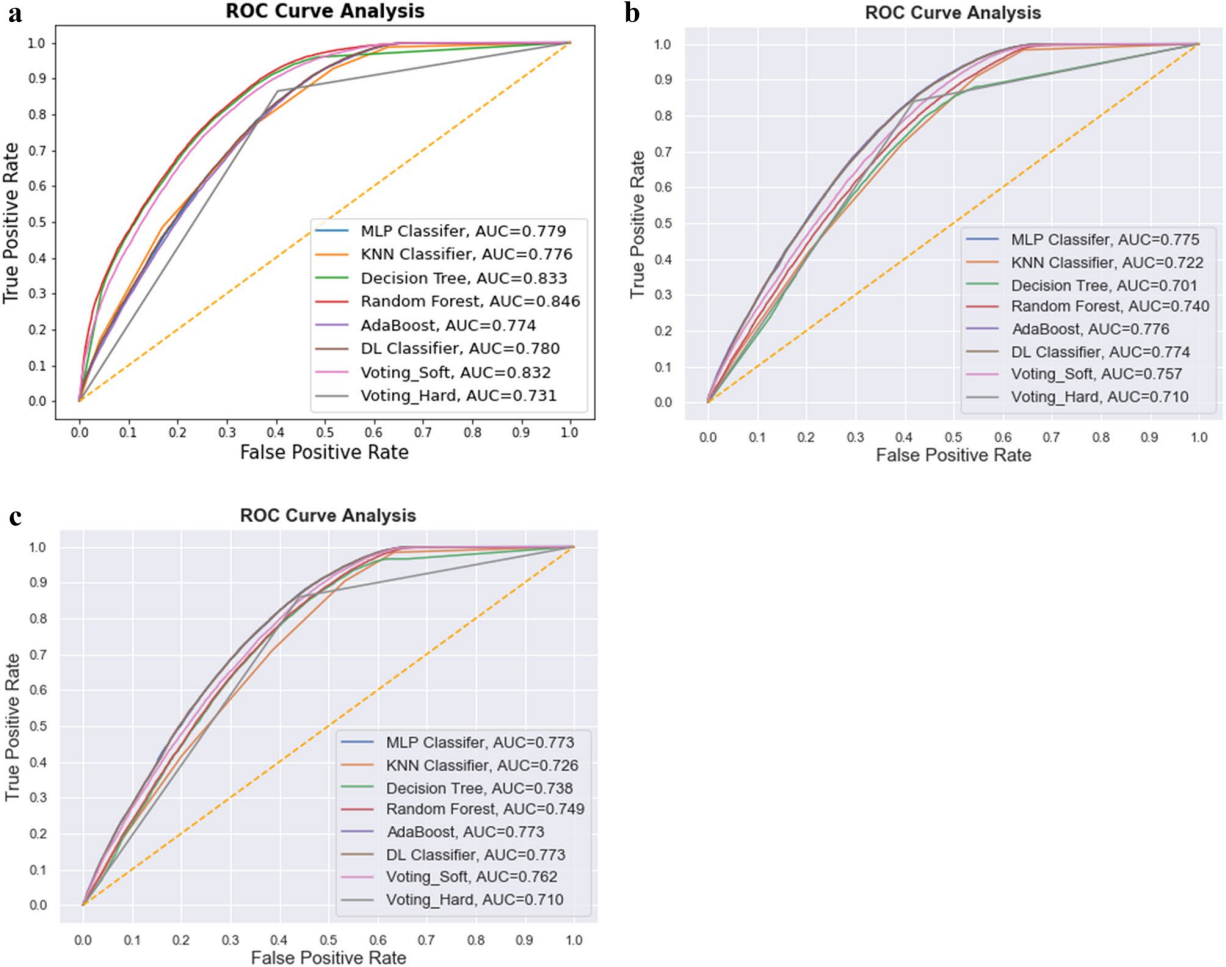
(**a**) ROC Curve Analysis of models trained without feature selection. (**b**) ROC Curve Analysis of different models in *With Feature Selection* setting, i.e., Linear SVC + Select From Model method. (**c**) ROC Curve Analysis of models trained with feature selection method, i.e., Linear SVC + Extra Trees classifier.

Table 2 explains that the ensemble learning models category results in better classification performance in the recall, F1-score, and ROC AUC scores. Random Forest scores the highest F1-score of 76.49%. The recall value achieved by random forest is noticeable among other trained models, i.e., 76%. Figure 4a Illustrates the ROC AUC curves of models trained without feature selection. The Random Forest has the highest AUC score of 84.6%.

### Results for with feature selection setting

#### *Method: linear SVC* + *SelectFromModel*

Table 3 describes that the ensemble learning models category has a better classification. The multi-layer perceptron and AdaBoost has the highest F1-score of 72.98%. AdaBoost, i.e., 77.60%, achieve the maximum ROC AUC score.

**Table 3:** Comparison between classification metrics of different models in *With Feature Selection* setting, i.e., Linear SVC + Select From Model.

	Model	Precision (%)	Recall (%)	F1-score (%)	ROC AUC score (%)
Machine Learning models	Multi-layer Perceptron	74	72	72.98	77.50
	K Nearest Neighbours	67	66	66.49	72.20
	Decision Tree	67	67	67.00	70.10
Deep learning model	DL Classifier	74	72	72.98	77.40
Ensemble Learning models	Voting—Hard classifier	73	71	71.98	71.70
	Voting—Soft classifier	71	70	70.49	75.70
	Random Forest	69	68	68.49	74.00
	AdaBoost	74	72	72.98	77.60

Figure 4b exhibits the ROC AUC curves of models trained in *With Feature Selection* setting, i.e., Linear SVC + Select From Model. In this method, AdaBoost has the highest AUC score of 77.60%. When these results are compared with previous results in Table 2, there is an impact of the Feature selection method, which decreased the overall performance in metrics such as ROC AUC scores, F1-scores, and recall.

#### *Method: linear SVC* + *Tree-based feature selection*

This method has used a Tree-Based feature extractor as Extra Trees Classifier, which is slightly different from random forests. Extra Trees classifier is different because it selects the random split to divide a parent node into two random child nodes.

Table 4 represents that again the Machine Learning based classification is better. 73.46% is the highest F1 score achieved by this feature selection method, which is less than the previous method. The maximum recall value achieved here is 72%, which is the same as the previous method. However, ROC AUC scores have been increased from the previous method except for deep learning classifiers and AdaBoost. Figure 4c. Portrays the ROC AUC curves of models trained in *With Feature Selection* setting, i.e., Linear SVC + Extra Tree classifier. AdaBoost, MLP, DL classifiers have the highest AUC score.

**Table 4:** Comparison between classification metrics of different models in *With Feature Selection* setting, i.e., Linear SVC + Extra Trees classifier.

	Model	Precision (%)	Recall (%)	F1-score (%)	ROC AUC score (%)
Machine Learning models	Multi-layer Perceptron	75	72	73.46	77.30
	K Nearest Neighbours	66	66	66.00	72.60
	Decision Tree	70	69	69.49	73.80
Deep learning model	DL Classifier	75	71	72.94	77.30
Ensemble learning models	Voting—hard classifier	73	71	71.98	71.00
	Voting—soft classifier	72	70	70.98	76.20
	Random Forest	71	70	70.49	74.90
	AdaBoost	74	71	72.46	77.30

When results from the above three methods are compared, it is clear and advisable that regular features without using any feature selection method, i.e., especially Random Forests (Ensemble Learning method), have better accuracy of 76.49% and an AUC score of 84.6%. Therefore, it is preferable to use this model in production for real-time results.

## Conclusion

In clinics, medical practitioners can provide counseling about live-birth based on their experience or their success rate of the fertility center, which can be inappropriate in some cases. This study will help both patients and medical practitioners make a concrete decision that depends on the tool predicting successful or unsuccessful IVF treatment based on a patient's natural measurable predictors. This tool will provide counseling to couples about their chances of getting live-birth to emotionally get prepared before going through costly and cumbersome IVF treatment. The Random Forest model without feature selection has shown the best result that achieved an AUC score of 84.60% and 76.49% F1-score compared with other models. However, it is not suggested to depend solely on this tool currently for decision making, as the data is received from a single source, so it is not generalized to all populations. The models were trained on limited factors, while several important factors, such as consumption of alcohol, smoking, caffeine consumption, hypertension, and other lifestyle factors that have a significant impact on predicting pregnancy, have not been considered due to the dataset's limitation.

The scope of the future works are that the data can be collected from various IVF clinics in different geographical locations to contain information on many races across the globe. Few parameters on individuals' lifestyle should be taken into consideration as these details indirectly affect fertility. AI performances can be improved if diverse data from various races and age groups is collected. A study can also be made regarding successful fertility that emphasizes each feature's importance in IVF. Different feature selection and dimensionality reduction methods can be used to improve model performances.

## Supplementary information

Supplementary Figure 1.
