# Supplementary material for: Machine learning predicts live-birth occurrence before in-vitro fertilization treatment
Source: Sci Rep. 2020 Dec 1;10:20925. doi: 10.1038/s41598-020-76928-z (PMC7708502; doi:10.1038/s41598-020-76928-z)
Supplement: Supplementary file 1 — Supplementary Figure 1. [file 41598_2020_76928_MOESM1_ESM.docx]

**Machine Learning predicts live birth occurrence before In-vitro fertilization treatment**

***Ashish Goyal^1^, Maheshwar Kuchana^1^, A.K. Prasada Rao^[[1]](#footnote-1)^*^1^***


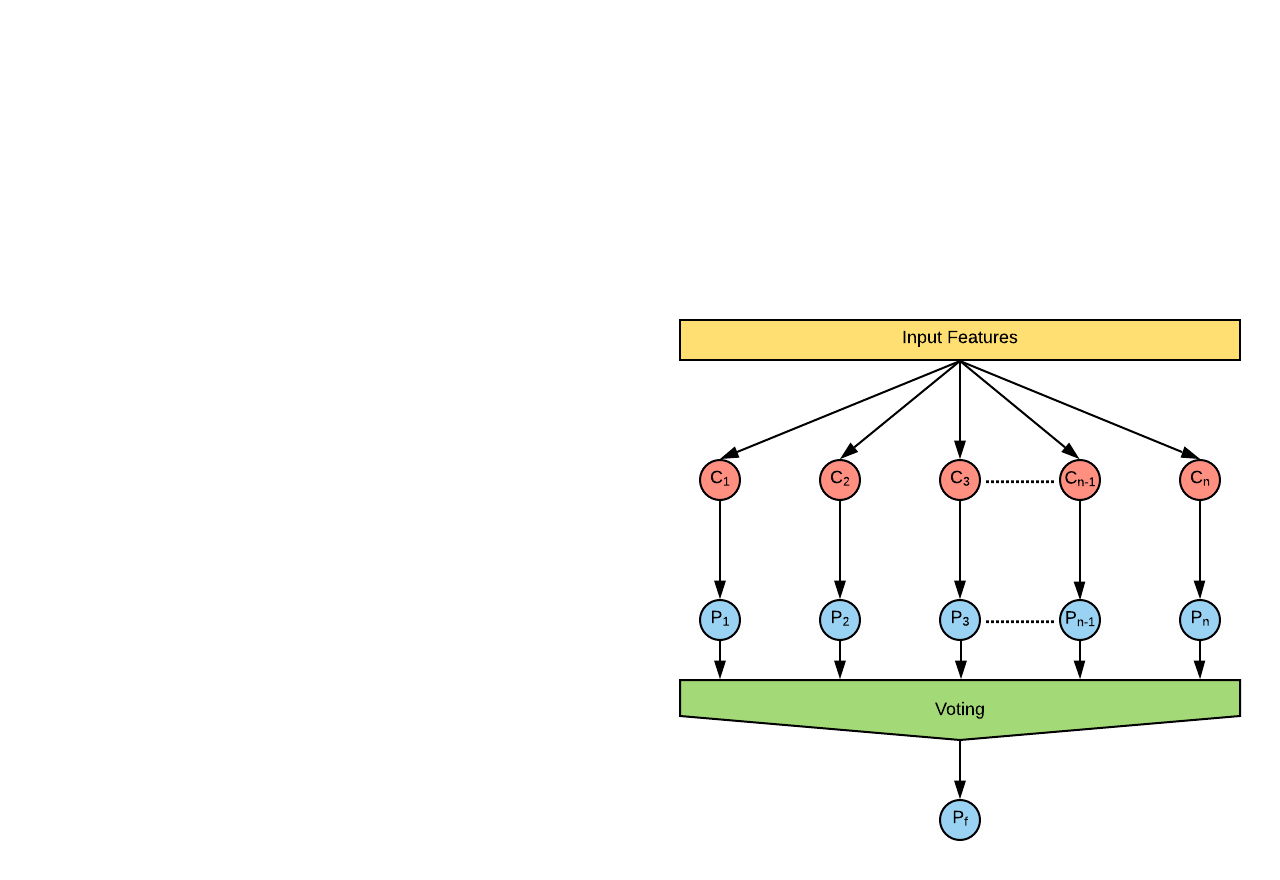


Voting Classifier

**Supplemental Fig.1**

1. * Corresponding author: Dr. A.K. Prasada Rao, Professor, BML Munjal University, Gurugaon, India – 122413. e-Mail: [akprasada@yahoo.com](mailto:akprasada@yahoo.com); Phone: +91-8295 96 3823 [↑](#footnote-ref-1)
